# Independent prognostic value of the triglyceride–glucose index and its incremental predictive contribution beyond traditional risk markers in acute heart failure: a retrospective cohort study

**DOI:** 10.3389/fcvm.2026.1785391

**Published:** 2026-05-29

**Authors:** Bolan Zhang, Bijian Wang, Lei Xia, Yaoyu Qi, Ying Xia, Binyan Lang, Yi Wang, Taidou Jiang, Yue Cao, Shuzhan Zheng

**Affiliations:** Department of Cardiology, Affiliated Hospital of Southwest Medical University, Luzhou, Sichuan, China

**Keywords:** cardiovascular disease, heart failure, NT-ProBNP, prognosis, triglyceride–glucose index

## Abstract

**Background:**

Risk stratification in patients hospitalized with acute heart failure remains challenging. Although N-terminal pro–B-type natriuretic peptide (NT-proBNP) is widely used for prognostic assessment, its predictive performance varies across clinical contexts. The triglyceride–glucose (TyG) index, a surrogate marker of insulin resistance, has been associated with adverse cardiovascular outcomes, but its prognostic relevance in acute heart failure is not well established.

**Methods:**

This single-center retrospective cohort study included 712 patients hospitalized with acute heart failure. TyG was calculated using fasting triglyceride and glucose levels at admission. The primary endpoint was a composite outcome of cardiovascular death or rehospitalization for heart failure. Cox proportional hazards models, restricted cubic spline analysis, threshold effect analysis, and receiver operating characteristic (ROC) curve analyses were performed.

**Results:**

During follow-up, 184 patients experienced the composite endpoint. In the fully adjusted Cox model, higher TyG levels were independently associated with an increased risk of the composite endpoint (HR = 1.74, 95% CI: 1.35–2.25, *P* < 0.001). When analyzed by quartiles, patients in the highest TyG quartile had a significantly higher risk compared with those in the lowest quartile (HR = 2.17, 95% CI: 1.34–3.50). Restricted cubic spline analysis demonstrated a significant nonlinear association, with a threshold identified at TyG = 9.659. ROC analyses showed that TyG had a discriminative ability comparable to NT-proBNP, while the combined model achieved a higher area under the curve than either marker alone.

**Conclusions:**

TyG is independently associated with adverse composite outcomes in patients

## 1 Introduction

Heart failure represents the terminal stage of various cardiovascular diseases and remains a leading cause of hospitalization and mortality worldwide (1). Patients with acute heart failure experience rapid disease progression, with substantially increased in-hospital mortality and a high risk of rehospitalization after discharge, imposing a considerable burden on both quality of life and healthcare systems (2). Previous studies have reported an in-hospital mortality rate of approximately 12% among patients hospitalized with acute heart failure, with 1-year rehospitalization and mortality rates reaching 45% and 22%, respectively (3). Given the heterogeneity and complexity of clinical presentations, accurate identification of high-risk patients and early risk stratification are critical for improving outcomes and optimizing resource allocation.

N-terminal pro-B-type natriuretic peptide (NT-proBNP), a biomarker reflecting ventricular wall stress and volume overload, is widely used for prognostic assessment in acute heart failure and has demonstrated value in predicting rehospitalization and mortality (4, 5). However, NT-proBNP levels are influenced by multiple factors, including age, body weight, and renal function, resulting in variable prognostic performance across different patient populations (5–11). In addition, although metabolic dysfunction plays an important role in the development and progression of heart failure, current risk stratification strategies incorporate few simple and readily available metabolic markers, which may limit their applicability in patients with metabolic abnormalities.

The triglyceride–glucose index (TyG), a simple surrogate marker of insulin resistance, integrates information from lipid and glucose metabolism. Emerging evidence has demonstrated close associations between TyG and adverse cardiovascular outcomes, including coronary artery disease, myocardial infarction, stroke, and chronic heart failure (12–20), suggesting a potential role of metabolic status in heart failure progression. Nevertheless, systematic evidence regarding the prognostic value of TyG in patients hospitalized with acute heart failure remains limited, and it is unclear whether TyG can complement or enhance risk discrimination beyond traditional biomarkers such as NT-proBNP.

Against this background, the present study aimed to evaluate the association between TyG and adverse outcomes in patients with acute heart failure, to assess its independent prognostic value in comparison with NT-proBNP, and to determine whether a combined model incorporating both markers could improve risk prediction. These findings may provide additional insight for early risk stratification in patients with acute heart failure.

## 2 Materials and methods

### 2.1 Study population

This was a single-center retrospective cohort study. Consecutive patients hospitalized with a diagnosis of newly diagnosed acute heart failure and acute exacerbation of chronic heart failure in the Department of Cardiology at the Affiliated Hospital of Southwest Medical University between January 2023 and August 2023 were screened for inclusion. Acute heart failure was diagnosed according to the 2022 ACC/AHA/HFSA Guideline for the Management of Heart Failure, based on typical clinical symptoms and signs, imaging evidence of pulmonary congestion or structural abnormalities, and age-specific diagnostic thresholds of NT-proBNP. Among 1,102 initially screened patients, individuals were excluded if they had severe valvular heart disease, hypertrophic cardiomyopathy, or other specific structural heart diseases; malignant tumors or autoimmune diseases; severe hepatic or renal dysfunction (defined as liver enzymes >3 times the upper limit of normal or estimated glomerular filtration rate <30 mL/min/1.73 m^2^); acute severe infection; recent major surgery or trauma; pregnancy or lactation; or missing key clinical data that precluded analysis. A total of 712 patients were ultimately included in the final analysis. The study protocol was approved by the Ethics Committee of the Affiliated Hospital of Southwest Medical University (approval number: KY2025014), and written informed consent was obtained from all participants.

### 2.2 Measurement and definition of TyG

Venous blood samples were collected in the fasting state on the morning of the day following admission. Fasting plasma glucose (FPG) and triglyceride (TG) levels were measured using a Mindray BS-2800M automated biochemical analyzer in the hospital's central laboratory. The TyG index was calculated according to the standard formula used in previous studies:

$$TyG\;=\ln\left(\frac{TG\;\times \;FPG}{2}\right)$$

### 2.3 Measurement of NT-proBNP

Blood samples for NT-proBNP measurement were obtained within 24 h of admission and prior to the initiation of anti–heart failure therapy. Plasma NT-proBNP levels were measured using a Lifotronic eCL8000I automated chemiluminescence analyzer, following the manufacturer's instructions.

### 2.4 Clinical data and laboratory assessment

Demographic characteristics (age and sex), lifestyle factors (smoking and drinking status), medical history (hypertension, diabetes mellitus, coronary artery disease, stroke, and atrial fibrillation), and in-hospital medication use were collected. Medications were recorded according to therapeutic categories and further classified into specific subtypes for coding and analysis. Glucose-lowering therapies included insulin and metformin; lipid-lowering therapy referred to statins; antihypertensive therapy mainly included calcium channel blockers; anticoagulant therapy included oral anticoagulants such as rivaroxaban and warfarin; antiplatelet agents included aspirin, clopidogrel, ticagrelor, and cilostazol; diuretics were categorized as loop diuretics or thiazide diuretics; and heart failure–modifying therapies included sodium–glucose cotransporter 2 inhibitors, angiotensin-converting enzyme inhibitors/angiotensin receptor blockers/angiotensin receptor–neprilysin inhibitors, β-blockers, and mineralocorticoid receptor antagonists. Laboratory parameters included lipid profiles, fasting plasma glucose, renal and liver function indices, uric acid, urea, hemoglobin, platelet count, D-dimer, and myocardial injury markers (cardiac troponin T, creatine kinase-MB, and myoglobin). Echocardiographic parameters included left atrial and left ventricular geometric measurements, ventricular volume indices, diastolic function parameters (E, A, e′, and E/e′), and left ventricular ejection fraction measured using the biplane Simpson method.

### 2.5 Covariate selection

Based on clinical relevance and previous literature, the following variables were selected as covariates for multivariable adjustment: age, sex, body mass index, drinking status; medical history of hypertension, diabetes mellitus, coronary artery disease, stroke, and atrial fibrillation; major medication categories during hospitalization (glucose-lowering agents, lipid-lowering agents, antihypertensive drugs, antiplatelet agents, diuretics, and heart failure–modifying therapies); and laboratory indices including low-density lipoprotein cholesterol, uric acid, and hemoglobin.

### 2.6 Outcome definition

All patients were followed up through review of electronic medical records supplemented by telephone interviews. The primary endpoint was the first occurrence of an adverse composite outcome, defined as cardiovascular death or rehospitalization for heart failure during the follow-up period. All outcome events were independently adjudicated by two investigators, and discrepancies were resolved by consensus.

### 2.7 Statistical analysis

All statistical analyses were performed using R software (version 4.4.2). Continuous variables are presented as medians with interquartile ranges and were compared using the Mann–Whitney *U*-test, while categorical variables are presented as counts and percentages and were compared using the *χ*^2^ test or Fisher's exact test, as appropriate. Cox proportional hazards regression models were used to evaluate the associations of TyG and the adverse composite outcome, with results reported as hazard ratios and 95% confidence intervals. Both univariable and multivariable models were constructed. Restricted cubic spline models were further applied to explore potential nonlinear relationships between TyG and the composite outcome, and a two-piecewise Cox regression model was used to assess threshold effects, with the inflection point automatically determined using a data-driven approach and model fit compared by likelihood ratio tests. Receiver operating characteristic curve analyses were performed to compare the discriminative ability of TyG, NT-proBNP, and their combination for predicting the composite outcome. All statistical tests were two-sided, and a *P* value <0.05 was considered statistically significant.

## 3 Results

### 3.1 Baseline characteristics

A total of 712 patients with acute heart failure were included in the analysis, among whom 184 experienced the primary composite endpoint of heart failure hospitalization or cardiovascular death during one year of follow-up. Compared with patients without events, those who experienced the composite endpoint had a higher prevalence of diabetes. Laboratory measurements showed that patients in the composite endpoint group had higher levels of fasting blood glucose, triglycerides, uric acid, blood urea nitrogen, serum creatinine, the triglyceride–glucose index (TyG), and NT-proBNP, as well as lower estimated glomerular filtration rate (all *P* < 0.01). Echocardiographic assessment demonstrated larger left ventricular end-diastolic and end-systolic dimensions, along with lower left ventricular ejection fraction and indices of diastolic function in patients with the composite endpoint (all *P* < 0.01). In addition, baseline use of glucose-lowering agents, diuretics, lipid-lowering agents, antiplatelet therapy, and ACEI/ARB/ARNI was more frequent in patients who experienced the composite endpoint (all *P* < 0.01). The main baseline differences between groups are summarized in Table 1.

**Table 1:** Baseline characteristics of patients according to the occurrence of the composite endpoint (heart failure hospitalization or cardiovascular death).

Variable	No composite endpoint *N* *=* *528*	Composite endpoint *N* *=* *184*	*P Value*
TyG	8.63 [8.33;8.99]	9.00 [8.50;9.51]	<0.001
Age	69.00 [58.00;77.00]	72.00 [63.75;79.00]	0.005
Sex, Male (%)	319 (60.42%)	105 (57.07%)	0.477
Height, cm	160.00 [155.00;166.00]	160.00 [155.00;167.00]	0.860
Weight, kg	61.00 [52.30;70.00]	60.00 [53.00;70.00]	0.873
BMI, kg/m^2^	23.90 [20.93;26.38]	23.78 [21.78;26.74]	0.373
Smoke status, *n* (%)	229 (43.37%)	77 (41.85%)	0.785
Drinke status, *n* (%)	177 (33.52%)	57 (30.98%)	0.588
Heart rate, beats/min	83.00 [71.00;96.00]	84.00 [72.00;96.25]	0.391
SBP, mmHg	126.00 [110.00;141.00]	124.50 [109.00;144.00]	0.909
DBP, mmHg	78.00 [68.75;90.00]	75.00 [68.00;85.00]	0.076
Hypertension, *n* (%)	320 (60.61%)	109 (59.24%)	0.811
Diabetes mellitus, *n* (%)	113 (21.40%)	85 (46.20%)	<0.001
CAD, *n* (%)	300 (56.82%)	121 (65.76%)	0.042
ACS, *n* (%)	193 (36.55%)	61 (33.15%)	0.459
Coronary Stent, *n* (%)	85 (16.10%)	43 (23.37%)	0.036
Stroke, *n* (%)	68 (12.88%)	30 (16.30%)	0.300
Atrial fibrillation, *n* (%)	117 (22.16%)	43 (23.37%)	0.813
Heart pacemaker, *n* (%)	12 (2.27%)	7 (3.80%)	0.290
Pulmonary embolism, *n* (%)	7 (1.33%)	5 (2.72%)	0.201
COPD, *n* (%)	53 (10.04%)	19 (10.33%)	1.000
Echocardiogram			
RVOT, mm	29.00 [27.00;32.00]	29.00 [27.00;31.00]	0.336
Aod, mm	32.00 [29.00;34.00]	32.00 [30.00;34.00]	0.941
LA, mm	37.00 [32.00;42.00]	39.00 [35.00;44.00]	0.001
AAO, mm	34.00 [31.00;37.00]	33.00 [31.00;36.00]	0.392
LVDd, mm	50.00 [46.00;59.00]	53.00 [47.00;62.00]	0.007
LVDs, mm	36.00 [30.00;46.00]	40.00 [32.00;51.00]	0.001
IVS, mm	10.00 [9.00;12.00]	10.00 [9.00;12.00]	0.959
LVPW, mm	10.00 [9.00;10.00]	10.00 [9.00;10.00]	0.236
MPA, mm	22.00 [21.00;24.00]	23.00 [21.00;25.00]	0.051
RV, mm	21.00 [20.00;23.00]	22.00 [20.00;24.00]	0.488
RA, mm	45.00 [40.00;52.25]	45.00 [40.00;54.00]	0.927
s, cm/s	6.00 [5.00;8.00]	6.00 [5.00;7.00]	0.004
e, cm/s	5.30 [4.00;7.00]	4.50 [4.00;6.00]	<0.001
a, cm/s	7.50 [5.00;9.00]	7.00 [5.00;8.93]	0.007
E/e	13.69 [10.13;19.62]	16.59 [11.65;22.00]	<0.001
E/a	0.72 [0.57;1.22]	0.67 [0.54;0.86]	0.024
LVEF, %	54.00 [41.00;62.00]	48.50 [39.00;59.00]	0.005
EDV, mL	119.00 [96.00;172.25]	136.00 [102.00;192.00]	0.006
ESV, mL	54.00 [36.00;94.00]	68.00 [42.00;119.25]	0.002
SV, mL	64.00 [52.75;79.00]	67.50 [53.00;81.00]	0.158
FS, %	28.00 [21.00;34.00]	24.50 [20.00;31.00]	0.003
Medication			
Hypoglycemic drugs, *n* (%)	68 (12.88%)	58 (31.52%)	<0.001
Insulin, *n* (%)	29 (5.49%)	20 (10.87%)	0.021
Metformin, *n* (%)	47 (8.90%)	43 (23.37%)	<0.001
Lipid lowering drugs, *n* (%)	174 (32.95%)	95 (51.63%)	<0.001
Statins, *n* (%)	174 (32.95%)	95 (51.63%)	<0.001
Antihypertensive drugs, *n* (%)	108 (20.45%)	40 (21.74%)	0.792
CCB, *n* (%)	108 (20.45%)	40 (21.74%)	0.792
Anticoagulant drugs, *n* (%)	63 (11.93%)	27 (14.67%)	0.404
OAC, *n* (%)	63 (11.93%)	27 (14.67%)	0.404
Antiplatelet drugs, *n* (%)	138 (26.14%)	80 (43.48%)	<0.001
Cilostazol, *n* (%)	19 (3.60%)	11 (5.98%)	0.242
Clopidogrel/Ticagrelor, *n* (%)	109 (20.64%)	65 (35.33%)	<0.001
Aspirin/Indobufen, *n* (%)	101 (19.13%)	61 (33.15%)	<0.001
Diuretics drugs, *n* (%)	124 (23.48%)	86 (46.74%)	<0.001
Loop Diuretics, *n* (%)	110 (20.83%)	82 (44.57%)	<0.001
Thiazide Diuretics, *n* (%)	21 (3.98%)	6 (3.26%)	0.831
HF drugs, *n* (%)	222 (42.05%)	112 (60.87%)	<0.001
SGLT2, *n* (%)	65 (12.31%)	49 (26.63%)	<0.001
ACEI/ARB/ARNI, *n* (%)	159 (30.11%)	85 (46.20%)	<0.001
Beta-blockers, *n* (%)	160 (30.30%)	80 (43.48%)	0.002
MRA, *n* (%)	124 (23.48%)	84 (45.65%)	<0.001
Blood test indicators			
FBG, mmol/L	5.62 [4.96;6.64]	6.76 [5.63;9.30]	<0.001
FBG, mg	101.16 [89.24;119.56]	121.68 [101.30;167.44]	<0.001
NT-proBNP, pg/mL	2381.00 [1182.00;5121.50]	5172.50 [2638.00;10386.00]	<0.001
LDL-C, mmol/L	2.56 [1.99;3.29]	2.59 [2.05;3.21]	0.470
HDL-C, mmol/L	1.20 [1.00;1.43]	1.15 [0.97;1.43]	0.272
TG, mmol/L	1.21 [0.94;1.68]	1.40 [1.01;2.04]	0.002
TG, mg	107.65 [83.28;148.85]	124.04 [89.26;181.19]	0.002
TC, mmol/L	4.30 [3.57;5.26]	4.42 [3.61;5.12]	0.562
HGB, g/L	130.00 [117.00;144.25]	128.00 [115.75;141.00]	0.112
PLT, 109/L	193.00 [156.75;239.25]	191.50 [155.00;244.00]	0.927
D-dimer, ug/mL	0.51 [0.30;1.06]	0.58 [0.37;1.31]	0.025
ALT, U/L	23.75 [15.70;39.18]	21.45 [14.67;37.40]	0.201
AST, U/L	28.50 [20.67;42.47]	26.15 [19.95;39.07]	0.134
TBIL, umol/L	13.70 [9.60;19.52]	13.50 [9.75;18.58]	0.835
UA, umol/L	378.35 [311.68;480.68]	423.60 [339.98;534.60]	0.007
UR, mmol/L	6.68 [5.32;8.73]	8.04 [6.43;10.38]	<0.001
Creatinine, umol/L	86.60 [71.90;106.85]	101.10 [76.95;127.30]	<0.001
GFR, mL/min/1.73 m^2^	73.55 [55.17;88.10]	60.65 [41.90;79.28]	<0.001
CK-MB, ng/m	2.57 [1.53;4.76]	2.57 [1.56;4.45]	0.896
cTnT, ng/mL	0.03 [0.02;0.19]	0.03 [0.02;0.16]	0.032
Myo, ng/mL	50.00 [33.83;89.35]	57.45 [37.22;97.41]	0.128

Data are presented as mean (standard deviation), median (interquartile range), or number (percentage). TyG, triglyceride–glucose index; BMI, body mass index; ACS, acute coronary syndrome; CAD, coronary artery disease; COPD, chronic obstructive pulmonary disease; HF, heart failure; RVOT, right ventricular outflow tract; AOd, aortic diameter; LA, left atrial dimension; AAO, ascending aorta diameter; LVEDD, left ventricular end-diastolic dimension; LVESD, left ventricular end-systolic dimension; IVS, interventricular septal thickness; LVPW, left ventricular posterior wall thickness; MPA, main pulmonary artery diameter; RV, right ventricular diameter; RA, right atrial dimension; EDV, end-diastolic volume; ESV, end-systolic volume; SV, stroke volume; FS, fractional shortening; LVEF, left ventricular ejection fraction.SGLT2i, sodium–glucose cotransporter 2 inhibitor; MRA, mineralocorticoid receptor antagonist; CCB, calcium channel blocker; OAC, oral anticoagulant; FBG, fasting blood glucose; NT-proBNP, N-terminal pro–B-type natriuretic peptide; LDL-C, low-density lipoprotein cholesterol; HDL-C, high-density lipoprotein cholesterol; TC, total cholesterol; TG, triglyceride; HGB, hemoglobin; PLT, platelet count; ALT, alanine aminotransferase; AST, aspartate aminotransferase; TBIL, total bilirubin; UA, uric acid; UR, urea; GFR, glomerular filtration rate; CK-MB, creatine kinase MB; hs-cTnT, high-sensitivity cardiac troponin T; Myo, myoglobin.

### 3.2 Association between TyG and the composite endpoint

The association between TyG and the composite endpoint was examined using Cox proportional hazards models (Table 2). When analyzed as a continuous variable, higher TyG levels were associated with an increased risk of the composite endpoint in the unadjusted model (HR = 1.95, 95% CI: 1.60–2.37). This association remained statistically significant after adjustment for age, sex, smoking status, and drinking status in Model 2 (HR = 2.14, 95% CI: 1.74–2.64). After further adjustment for clinical characteristics, comorbidities, medication use, and laboratory parameters in Model 3, the association was attenuated but remained statistically significant (HR = 1.74, 95% CI: 1.35–2.25).

**Table 2:** Association between the triglyceride–glucose index and the risk of the composite endpoint.

Variable	Model 1		Model 2		Model 3	
	HR (95% CI)	*P* value	HR (95% CI)	*P* value	HR (95% CI)	*P* value
TyG	1.95 (1.60, 2.37)	<0.001	2.14 (1.74–2.64)	<0.001	1.74 (1.35–2.25)	<0.001
Q1	Ref		Ref		Ref	
Q2	0.69 (0.41, 1.15)	0.156	0.74 (0.44,1.23)	0.241	0.67 (0.40,1.13)	0.132
Q3	1.27 (0.81, 1.98)	0.293	1.43 (0.90,2.24)	0.126	1.10 (0.68,1.77)	0.706
Q4	2.65 (1.79, 3.92)	0.000	3.20 (2.10,4.89)	0.000	2.17 (1.34,3.50)	0.002
*P* for trend	<0.001		<0.001		<0.001	

HR, hazard ratio; CI, confidence interval.

Model 1: Unadjusted.

Model 2: Adjusted for sex, age, smoking status, drinking status and BMI.

Model 3: Adjusted for sex, age, smoking status, drinking status, BMI, hypertension, diabetes, coronary artery disease, stroke, atrial fibrillation, hypoglycemic drug, lipid lowering, antihypertensive drug, anticoagulant drug, diuretics drug, heart failure drug, LDL-C, UA and HGB.

When TyG was categorized into quartiles, participants in the highest quartile (Q4) had a significantly higher risk of the composite endpoint compared with those in the lowest quartile (Q1) across all models, including the fully adjusted model (HR = 2.17, 95% CI: 1.34–3.50). A significant trend across increasing TyG quartiles was observed in all models (*P* for trend < 0.001).

### 3.3 Nonlinear association and threshold effect of TyG

**Figure 1:**
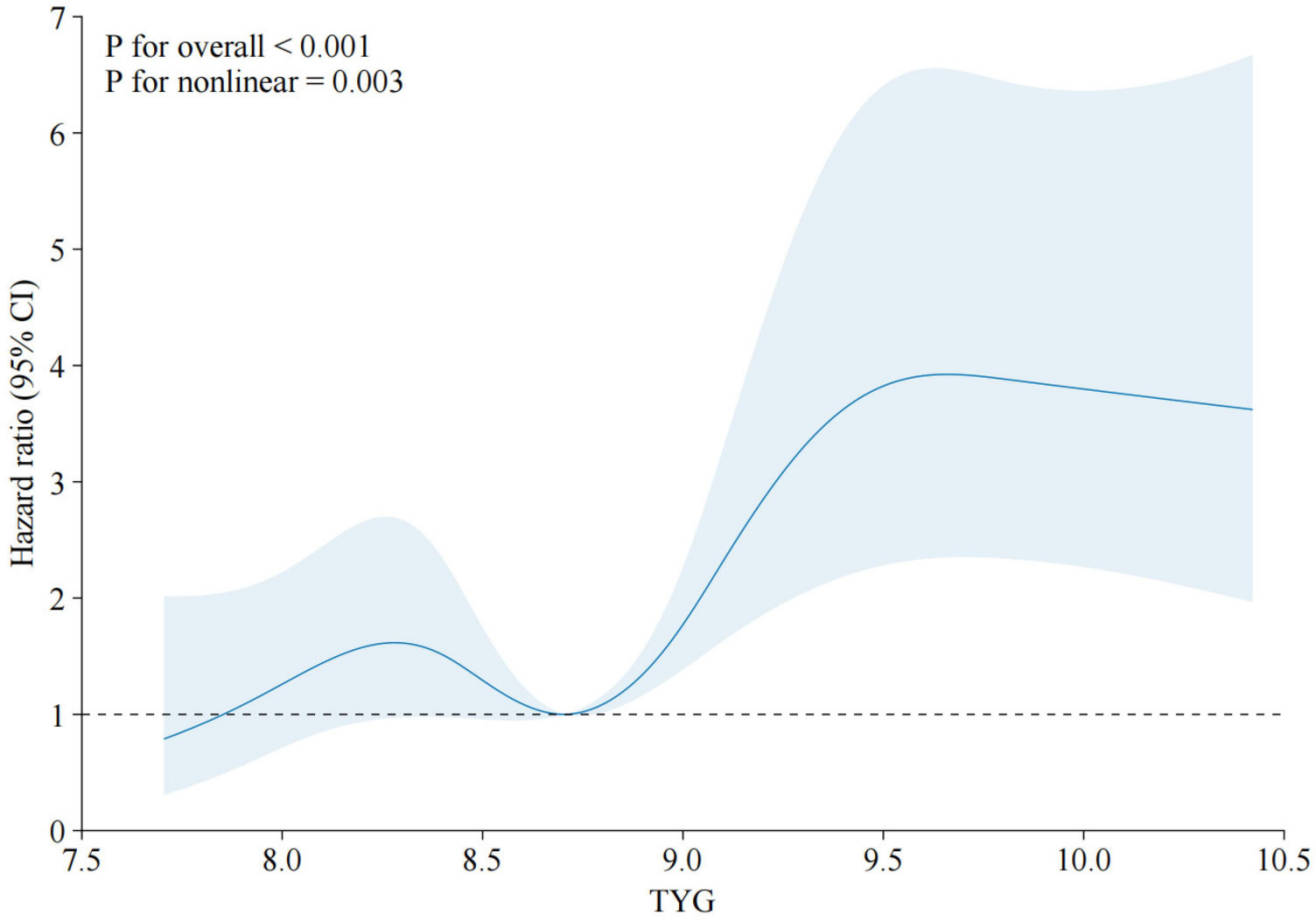
Restricted cubic spline analysis of the association between TyG and the composite endpoint in acute heart failure.

The potential nonlinear association between TyG and the composite endpoint was further evaluated using restricted cubic spline analysis (Figure 1). A significant overall association was observed (*P* for overall < 0.001), with evidence of nonlinearity (*P* for nonlinear = 0.003).

To further characterize this nonlinear relationship, a threshold effect analysis was conducted using a two-piecewise regression model, in which the inflection point was automatically identified. An inflection point of TyG was detected at 9.659 (Table 3). We have explicitly emphasized that the identified inflection point (TyG = 9.659) represents a statistical observation derived from the current dataset and should be interpreted as hypothesis-generating rather than clinically prescriptive. Below this threshold, higher TyG levels were associated with an increased risk of the composite endpoint (HR = 2.504, 95% CI: 1.717–3.652, *P* < 0.001), whereas no statistically significant association was detected in that range (HR = 0.728, 95% CI: 0.332–1.596, *P* = 0.429). The likelihood ratio test indicated that the two-piecewise model provided a better fit than the standard linear model (*P* = 0.008).

**Table 3:** Threshold effect analysis of TyG on the composite endpoint using a two-piecewise regression model.

Outcome	Effect	*P*
Model 1 Fitting model by standard regression	1.743 (1.353–2.246)	<0.001
Model 2 Fitting model by two-piecewise regression		
Inflection point	9.659	
<9.659	2.504 (1.717–3.652)	<0.001
>9.659	0.728 (0.332–1.596)	0.429
*P* for likelihood test		0.008

### 3.4 Subgroup analyses

**Figure 2:**
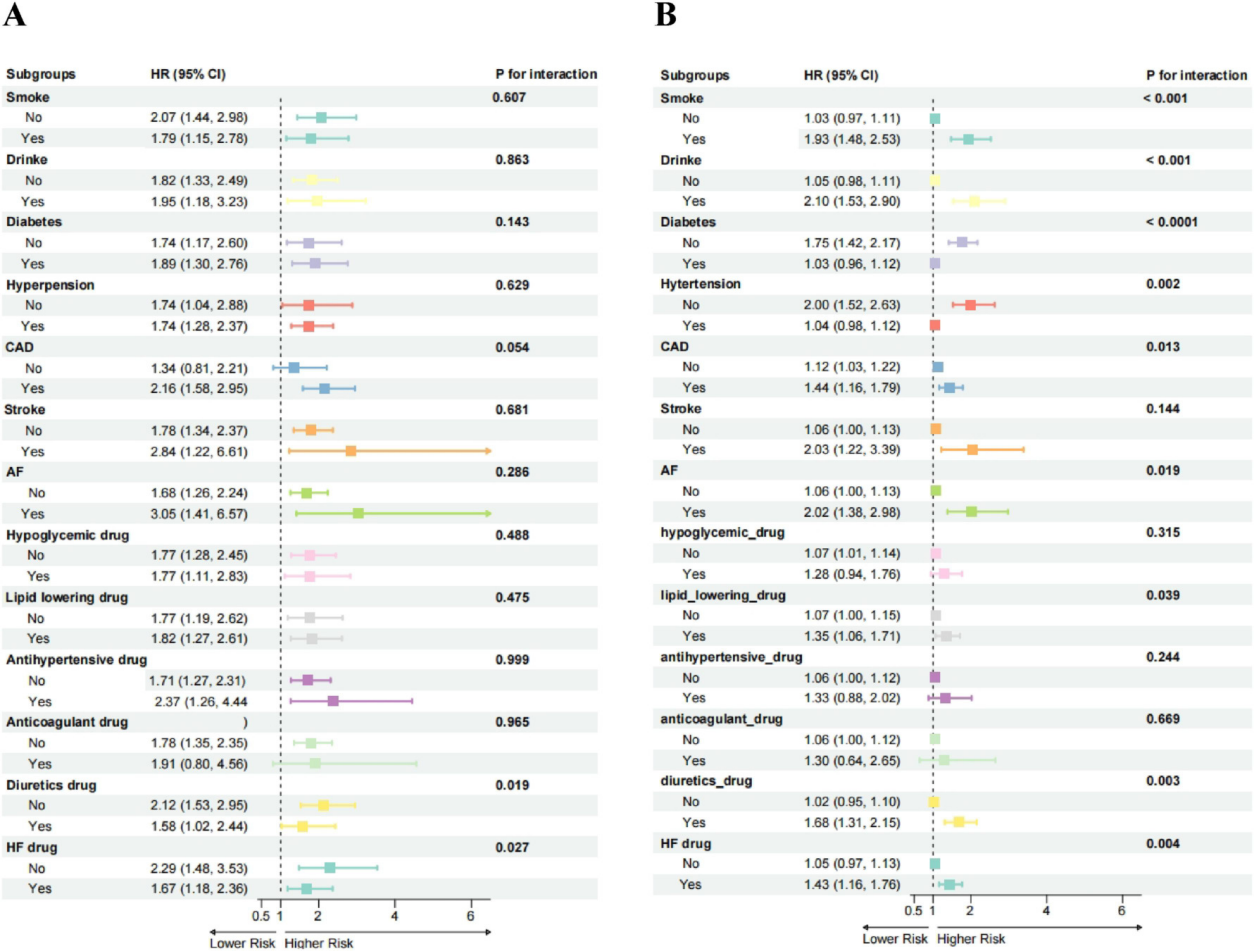
Subgroup analyses of the association between TyG **(A)** and NT-proBNP **(B)** with the composite endpoint.

Subgroup analyses were conducted to examine the associations of TyG and NT-proBNP with the composite endpoint across predefined clinical subgroups (Figure 2). As shown in panel A, the associations between TyG and the composite endpoint were generally consistent across subgroups defined by smoking status, drinking status, diabetes, hypertension, coronary artery disease, stroke, atrial fibrillation, and baseline medication use. Interaction tests for TyG were not statistically significant in most subgroups, although significant interactions were observed in certain subgroups. In panel B, the associations between NT-proBNP and the composite endpoint varied across subgroups, with statistically significant associations observed in some subgroups but not in others, and several subgroup variables showed significant interaction effects.

### 3.5 Receiver operating characteristic curve analysis

**Figure 3:**
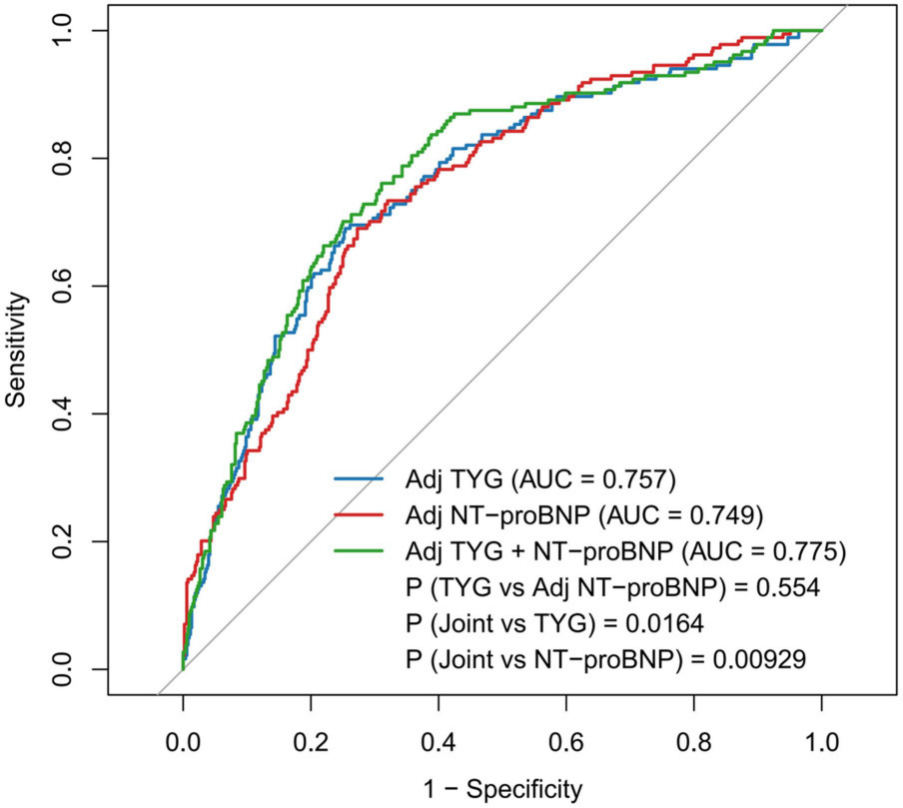
Receiver operating characteristic curves of TyG, NT-proBNP, and their combination for the composite endpoint.

Receiver operating characteristic curve analyses were performed to compare the discriminative performance of TyG, NT-proBNP, and their combination for the composite endpoint (Figure 3). When evaluated individually, NT-proBNP (AUC = 0.749) and TyG (AUC = 0.757) showed comparable discriminative ability, with no statistically significant difference between the two models (*P* = 0.554). When TyG and NT-proBNP were combined, the area under the curve increased to 0.775. Pairwise comparisons showed that the combined model achieved a significantly higher AUC than the model based on TyG alone (*P* = 0.016) and the model based on NT-proBNP alone (*P* = 0.009).

## 4 Discussion

In this single-center cohort of hospitalized patients with acute heart failure, the present study systematically evaluated the association between the triglyceride–glucose (TyG) index and 1-year adverse outcomes, defined as heart failure rehospitalization or cardiovascular death, and further explored its complementary role in relation to NT-proBNP. The main findings can be summarized as follows. First, TyG was significantly associated with 1-year adverse outcomes, and this association remained robust after multivariable adjustment. Second, restricted cubic spline analysis demonstrated a nonlinear relationship between TyG and heart failure outcomes, with a data-driven threshold identified. Third, when TyG was combined with NT-proBNP, the discriminative performance for adverse outcomes was significantly improved compared with either marker alone.

The nonlinear relationship observed in the present study was characterized by a flattening of the association at higher TyG levels. One possible explanation for this nonlinear pattern relates to the biological characteristics of insulin signaling and metabolic regulation. Previous studies have suggested that the TyG index may reflect a saturation phenomenon of insulin receptor activity within a certain range (approximately 8.5–9.0), beyond which further increases in insulin resistance may not proportionally translate into further metabolic or cardiovascular effects (21, 22). Under such conditions, the risk associated with TyG may plateau, resulting in an apparent attenuation of the association at higher values.

However, this interpretation should be approached with caution. An alternative and potentially complementary explanation is that patients with very high TyG levels represent a metabolically distinct subgroup, often characterized by overt diabetes, more severe systemic metabolic dysregulation, and complex comorbidity profiles. In such populations, the contribution of TyG as an isolated marker may be relatively diminished due to the dominance of other high-risk features, including hyperglycemia-related complications, inflammation, and competing clinical risks (23, 24).This shift in risk structure could partially account for the observed attenuation of the association at higher TyG levels.

Patients who experienced adverse outcomes had a higher prevalence of diabetes and coronary artery disease, along with elevated metabolic-related parameters, including fasting blood glucose, triglycerides, uric acid, and TyG, and more frequent use of glucose-lowering and lipid-lowering therapies. These findings are biologically plausible. Acute heart failure is accompanied by neurohormonal activation, systemic inflammation, and microcirculatory dysfunction, all of which can exacerbate insulin resistance. In turn, insulin resistance contributes to dysregulated lipid metabolism, enhanced oxidative stress, impaired myocardial energy utilization, and accelerated cardiomyocyte apoptosis (25–27). Elevated TyG levels therefore reflect an unfavorable metabolic environment and may be linked to a higher metabolic burden and energetic stress in the failing myocardium, thereby increasing susceptibility to heart failure decompensation, rehospitalization, and mortality. The role of metabolic dysfunction in heart failure progression has been increasingly recognized, and TyG, derived from routinely measured laboratory parameters, may sensitively capture deterioration in metabolic status that is not fully reflected by conventional biomarkers (28).

Compared with previous studies, the present findings extend existing evidence in several important aspects. Prior research has mainly focused on the association between TyG and chronic heart failure, coronary artery disease, or atherosclerotic cardiovascular risk (29–32). Some studies have reported an association between elevated TyG and increased mortality risk in patients with decompensated heart failure (33–35). However, systematic evidence regarding the prognostic relevance of TyG in hospitalized patients with acute heart failure remains limited. In the current study, TyG was not only significantly associated with 1-year adverse outcomes but also demonstrated generally consistent associations across a wide range of clinically relevant subgroups. In contrast, the associations observed for NT-proBNP showed greater heterogeneity across subgroups, with attenuated or nonsignificant associations in several clinical strata. In addition, receiver operating characteristic analyses showed that TyG and NT-proBNP had comparable discriminative ability when evaluated individually, whereas the combination of TyG and NT-proBNP resulted in a significantly higher area under the curve than NT-proBNP alone. Taken together, these findings suggest that metabolic information captured by TyG may complement traditional natriuretic peptide–based risk assessment.

From a clinical perspective, the present study highlights TyG as a simple, low-cost, and readily available metabolic indicator that may supplement established risk stratification strategies in acute heart failure. Although NT-proBNP remains the most widely used biomarker for heart failure diagnosis and prognostic assessment, its levels are influenced by age, sex, obesity, and renal function, which may lead to uncertainty in certain patient subgroups. In the present analysis, TyG remained associated with adverse outcomes in subgroups where NT-proBNP demonstrated weaker or more variable associations. Moreover, the combined use of TyG and NT-proBNP improved overall discrimination and risk reclassification for adverse outcomes. Because TyG is calculated entirely from routine fasting glucose and triglyceride measurements, its implementation does not increase testing burden and may be particularly feasible in real-world clinical practice and resource-limited settings.

Several limitations should be acknowledged. First, this was a single-center observational study, and causal inferences cannot be established. The TyG index was assessed at a single time point at admission, which does not capture dynamic changes in glucose and lipid metabolism during hospitalization or follow-up. As such, it remains unclear whether the observed associations reflect chronic metabolic status or acute stress-related alterations. Second, although a comprehensive set of clinical covariates was included, residual confounding from unmeasured or incompletely measured factors cannot be excluded. This study should be interpreted with appropriate caution when applied to patients with advanced renal dysfunction and stated that additional studies including patients with severe renal impairment are warranted to validate the clinical applicability of the TyG index in this high-risk population. These limitations warrant confirmation of the present findings in multicenter, large-scale prospective studies with repeated measurements.

## 5 Conclusion

The TyG index is independently associated with the composite endpoint in patients with acute heart failure. Compared with NT-proBNP alone, TyG provides additional prognostic information, and their combination further improves prediction of the composite endpoint.

## Data Availability

The raw data supporting the conclusions of this article will be made available by the authors, without undue reservation.
